# Reversible Self-Assembled Monolayers with Tunable Surface Dynamics for Controlling Cell Adhesion Behavior

**DOI:** 10.1021/acsami.2c12029

**Published:** 2022-09-08

**Authors:** Sing Yee Yeung, Yulia Sergeeva, Guoqing Pan, Silvia Mittler, Thomas Ederth, Tommy Dam, Peter Jönsson, Zahra El-Schich, Anette Gjörloff Wingren, Adam Tillo, Sabrina Hsiung Mattisson, Bo Holmqvist, Maria M. Stollenwerk, Börje Sellergren

**Affiliations:** † Department of Biomedical Sciences and Biofilms-Research Center for Biointerfaces (BRCB), Faculty of Health and Society, Malmö University, 205 06 Malmö, Sweden; ‡ Institute for Advanced Materials, School of Materials Science and Engineering, Jiangsu University, Zhenjiang, Jiangsu 212 013, China; § Department of Physics and Astronomy, University of Western Ontario, 1151 Richmond Street, London, Ontario, Canada N6A 3K7; ∥ Division of Biophysics and Bioengineering, Department of Physics, Chemistry and Biology (IFM), Linköping University, 581 83 Linköping, Sweden; ⊥ Division of Physical Chemistry, Department of Chemistry, Lund University, 221 00 Lund, Sweden; # ImaGene-iT AB, Medicon Village, Scheelevägen 2, 223 81 Lund, Sweden

**Keywords:** ECM mimic, reversible cell adhesion, dynamic multivalency, cell modulation, supported lipid bilayer

## Abstract

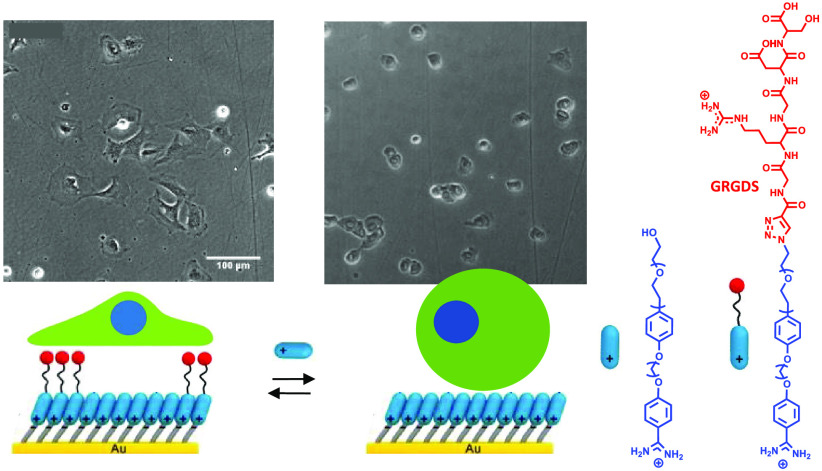

Cells adhering onto surfaces sense and respond to chemical and physical surface features. The control over cell adhesion behavior influences cell migration, proliferation, and differentiation, which are important considerations in biomaterial design for cell culture, tissue engineering, and regenerative medicine. Here, we report on a supramolecular-based approach to prepare reversible self-assembled monolayers (rSAMs) with tunable lateral mobility and dynamic control over surface composition to regulate cell adhesion behavior. These layers were prepared by incubating oxoacid-terminated thiol SAMs on gold in a pH 8 HEPES buffer solution containing different mole fractions of ω-(ethylene glycol)_2-4_- and ω-(GRGDS)-, α-benzamidino bolaamphiphiles. Cell shape and morphology were influenced by the strength of the interactions between the amidine-functionalized amphiphiles and the oxoacid of the underlying SAMs. Dynamic control over surface composition, achieved by the addition of inert filler amphiphiles to the RGD-functionalized rSAMs, reversed the cell adhesion process. In summary, rSAMs featuring mobile bioactive ligands offer unique capabilities to influence and control cell adhesion behavior, suggesting a broad use in biomaterial design, tissue engineering, and regenerative medicine.

## Introduction

Cells adhering onto a surface can sense and respond to a wide variety of chemical and physical features of the adhesive surface, including the molecular nature of the adhesive ligands, their local densities and mobilities, and the surrounding environment.^1−6^ These responses toward external cues regulate key cellular processes including tissue formation, cell survival, differentiation, migration, growth, and apoptosis. Integrins, the main cellular receptors for the extracellular matrix, have a key role in mediating these activities.^2,3^ The tripeptide Arg-Gly-Asp or RGD is one of the highly conserved peptide sequences present in the extracellular matrix (ECM) recognized by the integrins. Since its discovery, this peptide sequence and its variations have been integrated into and onto a variety of scaffolds to investigate the role of cell adhesion molecules during cell adhesion processes and fabrication of biomaterials for cell culture, tissue engineering, and regenerative medicine.^7−14^ The scaffolds for immobilizing bioactive peptides can be either static (biopolymers,^15^ self-assembled monolayers (SAMs)^16−18^) or dynamic (hydrogels,^19,20^ supported lipid bilayers (SLBs),^11^ host–guest-based assemblies,^19−22^ self-assembled peptide amphiphiles^23^). In terms of two-dimensional (2D) crystalline-like layers, the most well-studied cases are SAMs and SLBs.^10,11,21,24,25^ The former in combination with light-responsive,^16,21^ magnetic,^26^ or electrical-responsive functionalities,^17,27,28^ or host–guest-based chemistry,^19−22^ enable controllable surface properties for reversible cell adhesion albeit featuring only short-range dynamic properties. The latter, however, are characterized by their long-range lateral mobility, which is conducive to integrin clustering required for downstream signal transduction paths for cell growth and differentiation.^29^ These systems have recently gained attention for their tunable lateral dynamics in investigating cell adhesion behavior and differentiation in the absence of elastic components in static architectures.^11,30,31^ The downsides of SLBs as platforms for cell culture are their poor long-term stability, limited stability toward air exposure, and lack of stimuli responsiveness.^32,33^ As such, modulating cell adhesion behavior followed by subsequent cell release using 2D crystalline-like platforms is important for cell-based applications but an extreme challenge for material scientists.

**Figure 1:**
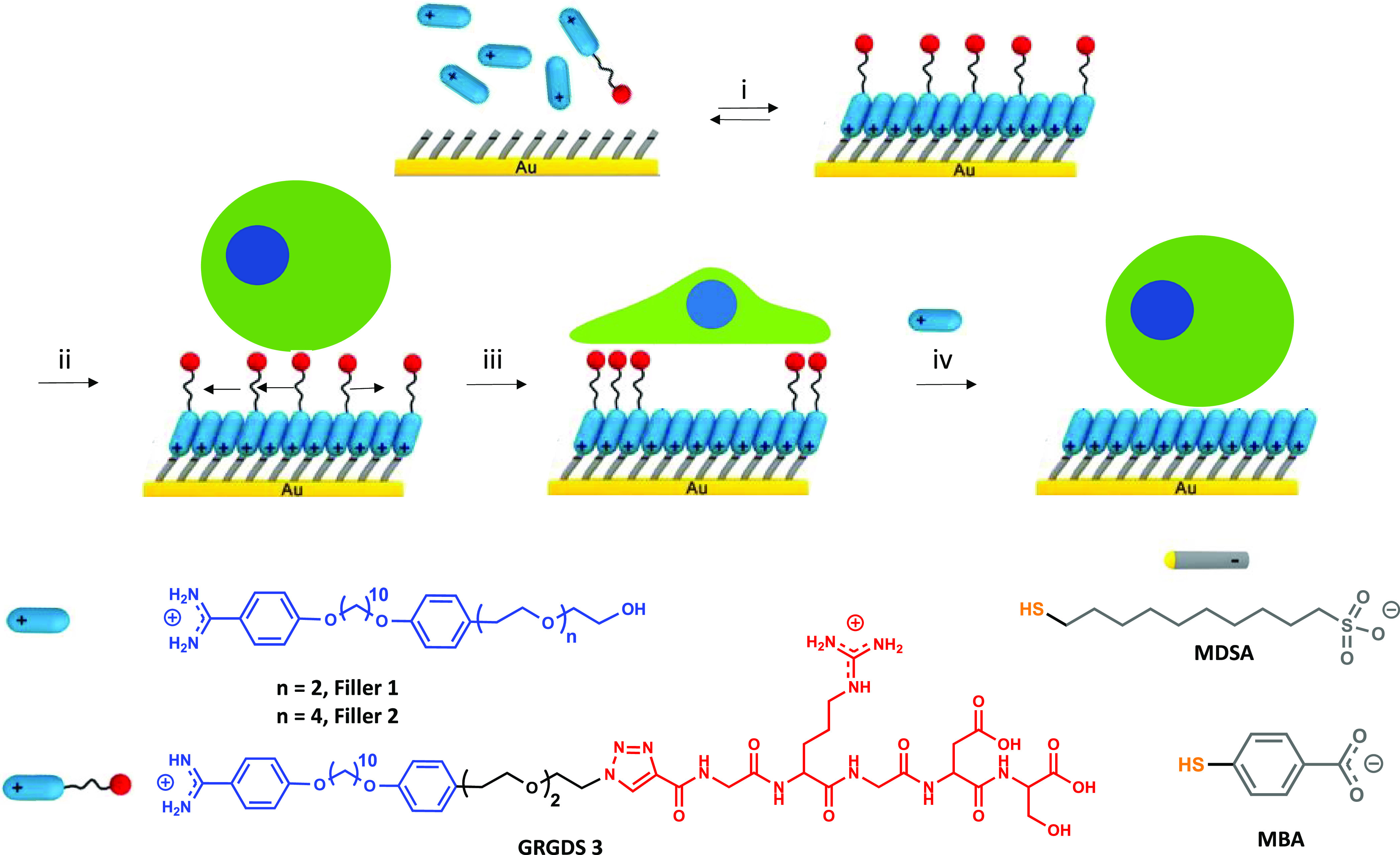
Schematic illustration of the modulation of cell adhesion behavior on reversible self-assembled monolayers (rSAMs) functionalized with a GRGDS peptide ligand. i. Incubation of 4-mercaptobenzoic acid (MBA) or 10-mercaptodecanesulfonic acid (MDSA) self-assembled monolayers (SAMs) in pH 8 HEPES buffer solution containing varying mole fractions, χ, of GRGDS 3 in filler 1 or 2, χ_GRGDS3_ = 0–0.25, followed by rinsing with pH 8 HEPES buffer. ii. Seeding of 3T3 fibroblasts on the rSAM surface. iii. Incubation for 5 hours. iv. Molecular exchange of GRGDS 3 with Filler 2 and complete cell detachment. Lower part: structural formulas of used molecules.

We recently reported on a robust and adaptable biosensing platform, reversible self-assembled monolayers (rSAMs), featuring strongly enhanced affinity and sensitivity toward proteins and viruses.^34^ This sensing system utilizes noncovalent amidinium–carboxylate ion pairs for assembly (on) and disassembly (off) of stable two-dimensional constructs, similar to lipid bilayers but with a simple preparation process and fast on/off rates. Benzamidine-terminated amphiphiles spontaneously assemble in neutral or alkaline aqueous solution onto alkanoic acid-functionalized thiol SAMs forming robust and ordered monolayers with tunable pH responsiveness. Layer thicknesses and order correlate with the molecular length of the amphiphile, which—beyond a certain length—features crystalline-like order and an odd–even chain length-related tendency to form bilayers. These layers are stable toward rinsing with neutral buffers and air exposure and resist exchange by common plasma proteins or charged surfactants while reducing nonspecific protein adsorption.^34−39^ Here, we demonstrate that a mixed rSAM functionalized with the pentapeptide GRGDS provides lipid bilayer-like lateral dynamics and the ability to modulate cell adhesion behavior. In addition, molecular exchange of the GRGDS-functionalized rSAMs with inert ethylene glycol filler amphiphiles enables dynamic reversal of cell adhesion (Figure 1).

## Results and Discussion

### Amphiphile Design and Synthesis

Optimization of RGD-functionalized surfaces for cell adhesion demands attention to parameters such as peptide sequence, number of ethylene glycol repeats in tether and filler molecule, ligand density, and lateral dynamics. This aims, inter alia, to reduce cell-surface nonspecific interactions and to avoid ligand–receptor steric hindrance. For instance, increasing ethylene glycol repeating units in the filler molecule typically decreases cell adhesion, whereas the lateral mobility of the ligand influences the area of adhered cells and focal adhesion formation.^4,40−42^ In this study, we compared GRGDS-functionalized bolaamphiphile 3 (GRGDS 3) in combination with ethylene glycol (EG)-functionalized amphiphiles with either two or four EG repeat units (Filler 1 and 2, respectively) to form stimuli-responsive layers (Figure 1). GRGDS 3, Filler 1, and 2 were synthesized as described in the Supporting Information and as previously reported.^38^

### rSAM Formation and Characterization

rSAMs functionalized with GRGDS 3 were prepared as previously reported^39^ by 18 h incubation of MBA or MDSA SAMs in pH 8 HEPES buffer solution containing different mole fractions, χ, of GRGDS 3 and Filler 1 or 2 (χ_GRGDS3_ = 0–0.25). Formation, structure, and dynamic properties of the adsorbed films were investigated by *in situ* ellipsometry (ISE), infrared reflection–absorption spectroscopy (IRAS), and fluorescence recovery after photobleaching (FRAP), respectively, following previously reported protocols.^38,39^

ISE measures refractive index- and film thickness-sensitive changes of the polarization when light is reflected from a surface. The ellipsometric angles Δ and Ψ correlate with the phase shift and amplitude ratio of the s- and p-components of the reflected light and are used to estimate film thickness and mass in real time. We first investigated the adsorption behavior of pure Filler 2 (χ = 0) and Filler 2 mixed with GRGDS 3 at χ_GRGDS3_ = 0.10 and χ_GRGDS3_ = 0.25 on the pure MBA and pure MDSA anchor SAMs (Figure 1).

Figure S1 (see the Supporting Information) shows the average film thicknesses during adsorption of the amphiphiles dissolved in pH 8 HEPES buffer (total concentration = 50 μM). After injection, the film thicknesses increase steeply, and within 1 min, films featuring thicknesses corresponding to monolayers are formed. The agreement between the thicknesses measured after rinsing and the amphiphile molecular lengths as well as the significant increase in film thicknesses when introducing GRGDS 3 at different mole fractions indicates the presence of mixed rSAMs of densely packed amphiphiles oriented perpendicularly to the surface.

The structure and composition of the films were subsequently investigated by infrared reflection–absorption spectroscopy (IRAS). The IRAS spectra of the mixed rSAMs (Figure S2) were compared with respect to features informative of layer stoichiometry as well as order and orientation of the amphiphile molecules. Significant IRAS peaks of the anchor SAM (MBA or MDSA) and the two-component rSAMs could be identified (Table S1). As a general observation, the sharp and intense aromatic C=C stretch signals of the bolaamphiphiles (1611 cm^–1^ on MDSA and 1609 cm^–1^ on MBA) and weak C–H out-of-plane bending signal at 840 cm^–1^ indicate the presence of ordered layers of amphiphiles with a near upright orientation. The intensities of several signals were affected by introducing GRGDS 3. Figure S3 shows average signal intensities and integrals (*n* = 3) for key signals reflecting the mixed rSAM stoichiometry. On the MDSA SAM, nearly linear increases in signal intensities with increasing χ_GRGDS3_ were observed for the bands assigned to the peptide ligand, *i.e.*, the amide A (3250 cm^–1^, Figure S3C) and the amide I bands (Figure S3A). This was accompanied by a smaller increase in the intensity of signals in the region 750–950 cm^–1^, which we assign to overlapping bands of the peptide amide V (N–H out-of-plane bending) vibration at 800 cm^–1^ and the aromatic C–H out-of-plane mode at 840 cm^–1^. Collectively, these observations support an unbiased incorporation of GRGDS 3 into the rSAM reflecting the solution stoichiometry (χ_GRGDS3_). As suggested by the concomitant decrease of the aromatic C=C (1611, 1512, 1495 cm^–1^) and aryl–alkyl ether (1248 cm^–1^) stretch signals, increasing the GRGDS ligand density leads to a slightly more tilted arrangement of the rSAM amphiphiles. Meanwhile, the intense MDSA sulfate S=O stretch band at 1046 cm^–1^ was not affected by the mixing ratio. Turning to the MBA-SAM, the rSAM showed a more complex behavior (Figures S2B and S3B,D). The apparent increase in the amide I band (Figure S2B) integral was associated with a large spread, presumably caused by the overlapping MBA C=O stretch signal at 1720 cm^–1^ and its shift to lower frequency with increasing hydrogen bonding interactions. However, additional confirmation for the presence of the peptide ligand was the increased intensity of the amide V band at 800 cm^–1^. Increasing χ_GRGDS3_ also led to an increase in the intensity of the vibrations at 1611 and 1260 cm^–1^ with dipoles oriented along the aryl 1,4 length axis and a decreased intensity of the aryl CH out-of-plane bending mode at 840 cm^–1^. Contrary to the MDSA system, this indicates a trend toward a more upright orientation of the aryl groups.

FRAP was then used to gain insight into the rSAMs dynamic properties (Figure S4). Dye-doped rSAMs of Filler 1 with χ_GRGDS3_ = 0.10 anchored on the MBA and the MDSA SAMs were examined by following the recovery process in the bleached regions. The monolayers featured diffusivities in the range of 0.4–0.8 μm^2^/s and a 0.1–0.3 immobile fraction.

### Influence of Ligand Presentation and Density on Fibroblast Adhesion

**Figure 2:**
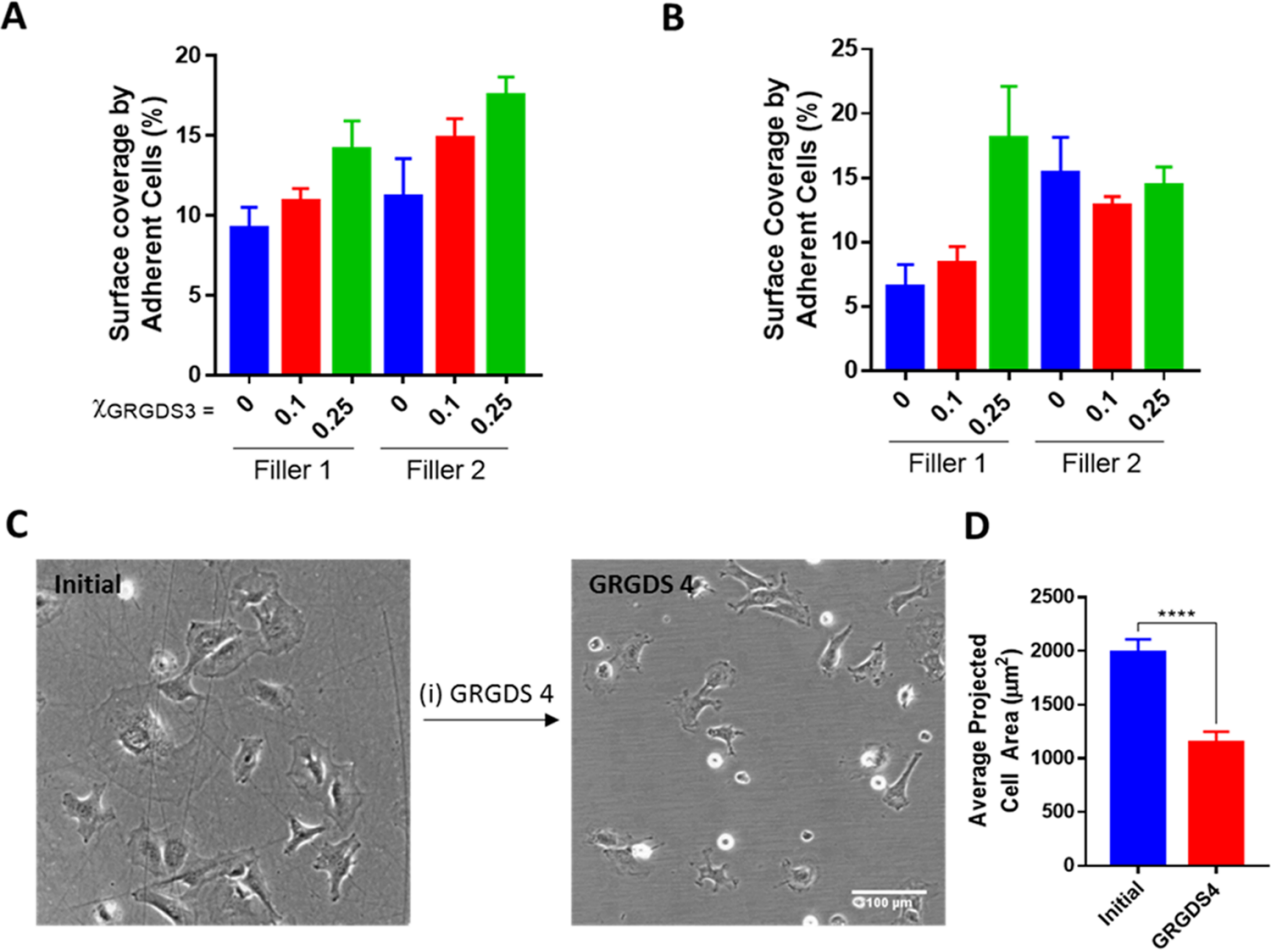
Effect of GRGDS 3 density, filler amphiphile, and anchor SAM on MC3T3-E1 adhesion. Percentage surface coverage by adherent MC3T3-E1 (%) as presented in brightfield micrographs (Figure S4) of MC3T3-E1 after culture for 5 h on (A) MBA SAMs or (B) MDSA SAMs modified with different mole fractions of GRGDS 3 in Filler 1 or 2, χ_GRGDS3_ = 0–0.25. (C) Representative brightfield micrographs of MC3T3-E1 after culture for 5 h on MBA SAMs modified with χ_GRGDS3_ = 0.25 (left) and after incubating in 100 μM GRGDS 4 for 2 h (right). (D) Specificity of GRGDS-integrin binding for cell adhesion determined by calculating the average projected cell area per cell in panel (C) (****p < 0.0001).

With the successful incorporation of GRGDS 3 into the amphiphile layers evidenced by ISE and IRAS, we evaluated the surfaces’ ability to regulate cell adhesion based on (a) the nature of the anchor SAM, (b) the mole fraction of GRGDS 3 in the assembling solution, and (c) the molecular length of the filler. Two types of gold substrates were used for this purpose. We first implemented homemade gold-coated 24-well cell culture plates^43^ and brightfield microscopy to investigate cell coverage and morphology. Phalloidin staining was used to visualize the cells’ F-actin structure. A quantitative assessment of the average projected area and shape of adhered cells was carried out. With the exception of Filler 2 rSAMs on MDSA, the coverage of adhered cells increased with increasing χ_GRGDS3_, *i.e.*, with increasing GRGDS 3 ligand density (Figures 2 and S5). This result agrees with reports in the literature on cell adhesion on RGD-functionalized SLBs.^30,31^

**Figure 3:**
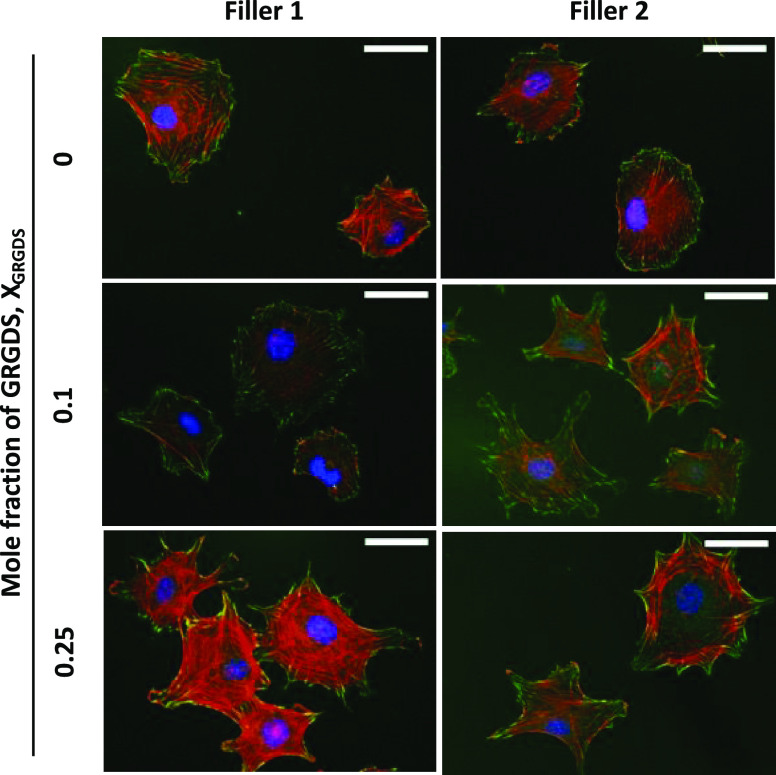
Double immunofluorescence labeling. Representative images illustrate the effect of GRGDS 3 density and filler amphiphiles on the morphology of MC3T3-E1 cells adhered to rSAMs anchored on MBA-SAMs. The labeling used to visualize the cells are nucleus (DAPI: blue), focal adhesions (phospho-paxillin: green), and actin filaments (phalloidin: red). The images were recorded 5 h after seeding. Scale bars = 50 μm.

To prove that the increased coverage was a result of primary interactions between cell surface integrins and the RGD ligands, we first attempted staining of the focal adhesion points by immunofluorescence using tyrosine-phosphorylated paxillin (p-paxillin). However, presumably due to gold-induced quenching, no fluorescence signal could be observed. Taking another approach, we tested whether the cells could be detached by ligand displacement. Thus, the adhered cells on the rSAM-coated MBA-SAM (χ_GRGDS3,Filler2_ = 0.25) were exposed to free ligand GRGDS 4 solution at a concentration of 100 μM (see Scheme S1). After 2 h, a 50% decrease in average projected cell area was observed (Figure 2D); hence, the introduction of GRGDS 4 diminishes cell adhesion. In addition to the results in Figure 2 (vide infra), this suggests that incorporation of GRGDS 3 promotes specific RGD-integrin-mediated cell adhesion.^44^ To overcome the fluorescence quenching problem of the immunofluorescence staining, we repeated the experiments using microscope coverslips coated with ultrathin (*d* = 10 nm) gold films. The rSAM preparation and cell culturing were carried out in an identical manner as on the well plates but using only MBA as anchor SAM. Imaging was then performed by inverted widefield fluorescence microscopy. Cells were cultured in triplicate wells, and the experiment was repeated twice per batch on two different cell batches. As shown in Figure 3 (Figure S6A), most of the cells seeded on the Filler 1 rSAMs possessed round-shaped morphology with dot-like radially distributed focal adhesions (FAs) featuring only a small fraction of FAs at the cell edges. Turning to the rSAM with χ_GRGDS,Filler1_ = 0.1, this surface showed a higher fraction of cells displaying elongated FAs at the cell periphery, although still with a low amount of actin bundles. The cells plated on χ_GRGDS,filler1_ = 0.25 showed larger protrusion of actin stress fibers coaligned with the FAs mostly presented at the cell edges. A similar behavior was observed for the rSAMs made of Filler 2 (Figures 3 and S6B). Interestingly, the cells plated on the χ_GRGDs,filler2_ = 0.1 showed an increased spreading compared to those plated on χ_GRGDs,Filler1_ = 0.1. For now, we tentatively ascribe this effect to higher mobility of amphiphiles in the former rSAM. Indeed we previously showed that rSAM stability decreases with an increasing number of EG repeats.^38^

### Influence of Lateral Dynamics on Fibroblast Morphology

**Figure 4:**
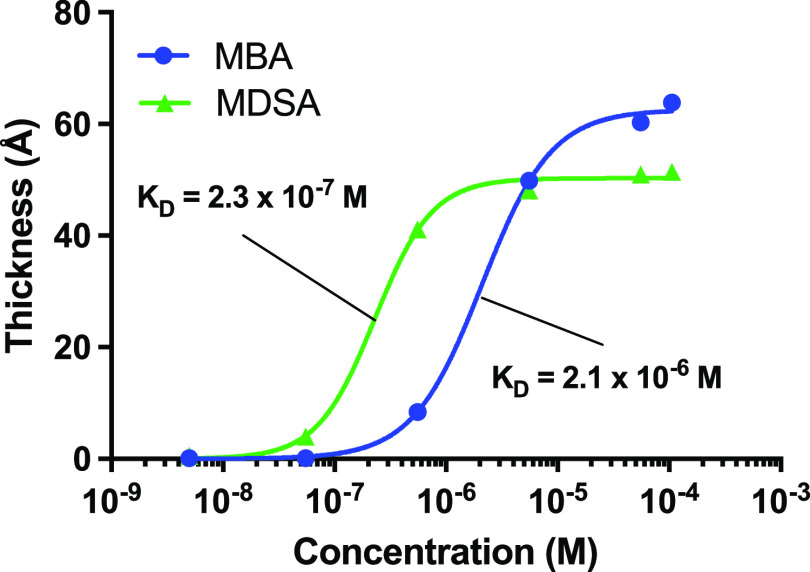
Adsorption isotherms of Filler 2 on MBA (blue) and MDSA (green)-SAMs. The lines are fits to the Hill equation yielding the respective *K*_D_ values.

As one of the outstanding features of rSAMs is their long-range lateral fluidity mimicking natural biomembranes,^39^ the question arose whether this characteristic would be able to influence cell morphology in a controllable manner. Our hypothesis was that the two SAMs (MBA and MDSA) would anchor the rSAMs more or less firmly leading to different lateral mobilities^45^ of the RGD amphiphiles with a potential impact on receptor clustering. To investigate this, we first titrated the MBA- and MDSA SAMs with Filler 2 and measured the equilibrium film thickness with ISE (Figure 4). Fitting the resulting adsorption isotherms with the Hill equation,^46^ and comparing the corresponding dissociation constants (K_D_) showed that Filler 2 associated more firmly with the MDSA (*K*_D_ = 2.3 × 10^–7^ M) than to the MBA (*K*_D_ = 2.1 × 10^–6^ M) SAM. This is in agreement with the degree of ionization of the two SAMs, with MDSA (p*K*_a_ = −2.6) being a stronger acid than MBA (p*K*_a_ = 4.1), thus resulting in a SAM displaying a higher charge density. The enhanced charge–charge interactions in close-packed SAMs, however, can shift the p*K*_a_ values upward with several units and thereby suppress ionization.^47,48^ To investigate whether the different affinities led to different ligand mobilities, we investigated the rSAMs by FRAP. As seen in Figure S4, the amphiphiles appeared to be somewhat less mobile on the MDSA SAM—as reflected in their lower average diffusion constants (Figure S4A) and higher average immobile fraction (Figure S4B)—although these effects were not statistically verifiable.

**Figure 5:**
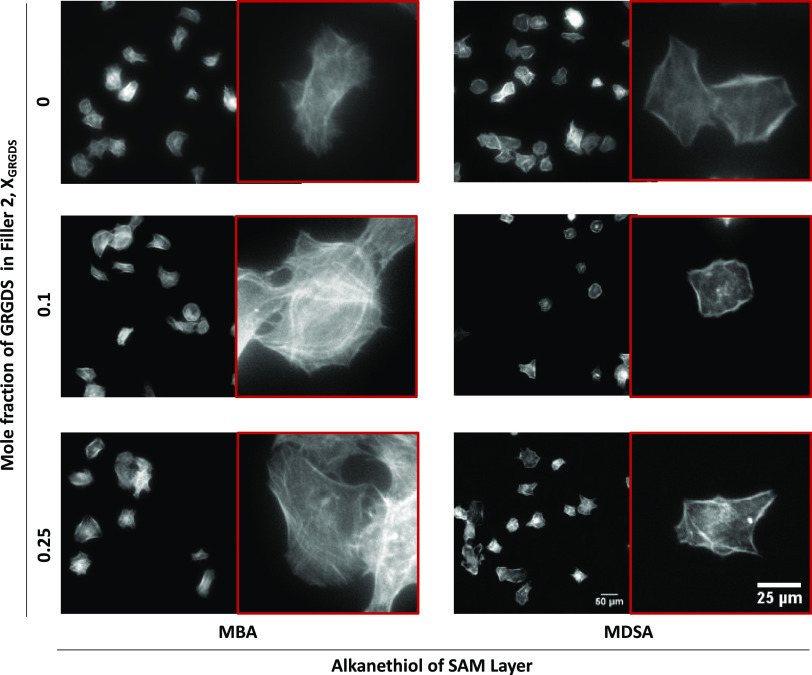
Differences in cell morphology on MBA- or MDSA-anchored rSAMs. Fluorescence micrographs of actin-stained MC3T3-E1 after culture for 5 h on MBA or MDSA SAMs modified with varying mole fractions of GRGDS 3 and Filler 2, χ_GRGDS3,Filler2_.

**Figure 6:**
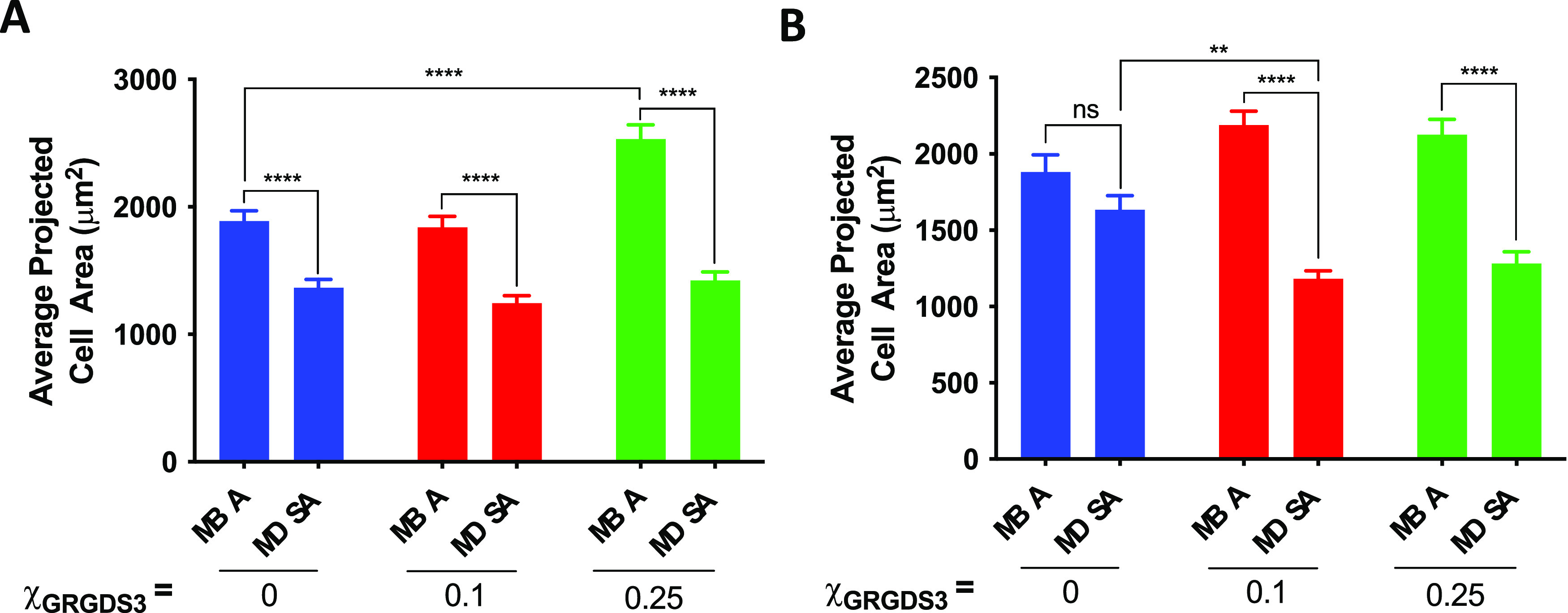
Differences in cell morphology on MBA- or MDSA-anchored rSAMs. (A) Average projected cell area of MC3T3-E1 attached on surface modified with varying mole fractions of GRGDS 3 and Filler 1, χ_GRGDS3,Filler1_ on either MBA or MDSA SAMs described in Figure S7. (B) Average projected cell area of MC3T3-E1 attached on the surface with varying mole fractions of GRGDS 3 and Filler 2, χ_GRGDS3,Filler2_ on either MBA or MDSA SAMs in Figure 5 (****p < 0.0001; **p < 0.01).

We then investigated the average projected cell area and cell shape of the adhered cells in more detail. For this purpose, the cells adhered on the rSAMs were stained with FITC-phalloidin to visualize the F-actin structure and imaged by fluorescence microscopy while comparing the influence of the type of anchor SAM (Figure 5). On the MBA-SAMs, the adhered fibroblasts consistently presented larger average projected cell areas than on the MDSA SAMs, regardless of filler length and density of GRGDS 3 (Figures 5 and 6, and S7). We tentatively ascribe this to the aforementioned mobility difference between the two rSAMs, which is in line with the observations by Kocer et al. with respect to cell adhesion and SLB mobility.^31^

Examination of the actin-stained cells on the MDSA-anchored rSAMs confirmed these findings with distinct differences in cell morphology as compared to the cells on the MBA-anchored rSAMs. The adhered cells on the MDSA rSAMs displayed a smaller average cell area than those on the latter. In the absence of the GRGDS 3 in the layer, the average cell area was sensitive to the length of the ethylene glycol chain of the filler. For instance, cells on MDSA anchored Filler 1 showed a 28% smaller average cell area as compared to those on the MBA-anchored filler molecule, whereas the use of Filler 2 led to no apparent differences (Figures 6 and S7). With the inclusion of GRGDS 3 in the rSAMs, the choice of oxoacid on the SAM, the type of filler used, and the GRGDS 3 density influenced the average projected cell area. For example, with Filler 1, no distinct differences were observed with the cells adhered on the MDSA-anchored rSAMs, whereas on MBA, we observed an increase in average cell area for χ_GRGDS3_ = 0.25 with respect to the surface without GRGDS 3 (Figure 6A). With Filler 2, there was no distinct difference between the cells adhered on rSAMs of different GRGDS 3 densities on MBA SAMs, whereas a decrease in average cell area was observed at χ_GRGDS3_ = 0.1 and 0.25 on the MDSA-anchored rSAMs as compared to the surface without GRGDS 3 (Figure 6B).

### Reversible Cell Adhesion via Molecular Exchange

**Figure 7:**
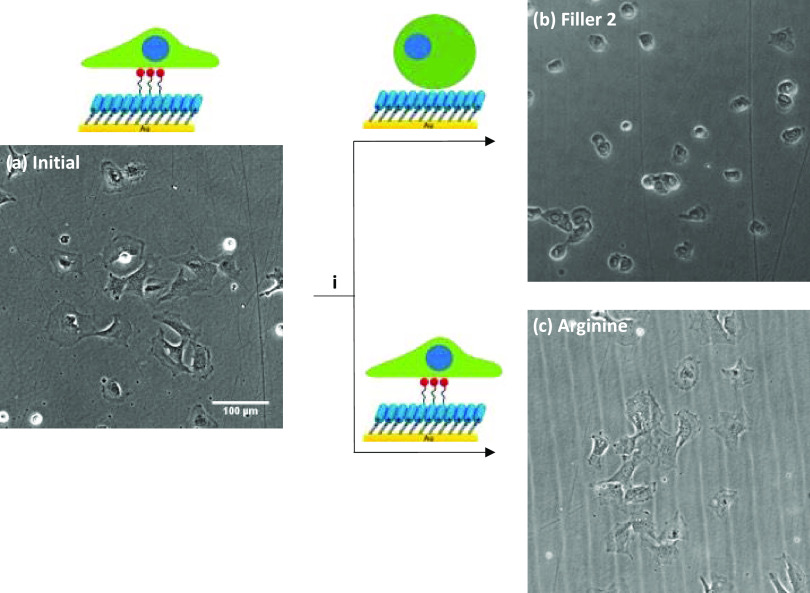
Reversible cell adhesion induced by the molecular exchange. Representative brightfield micrographs (a) initial of MC3T3-E1 after culture for 5 h on MBA modified with χGRGDS3 = 0.25 in Filler 2 and (i) 30 min after addition of 100 μM of (b) Filler 2 and (c) l-arginine.

**Figure 8:**
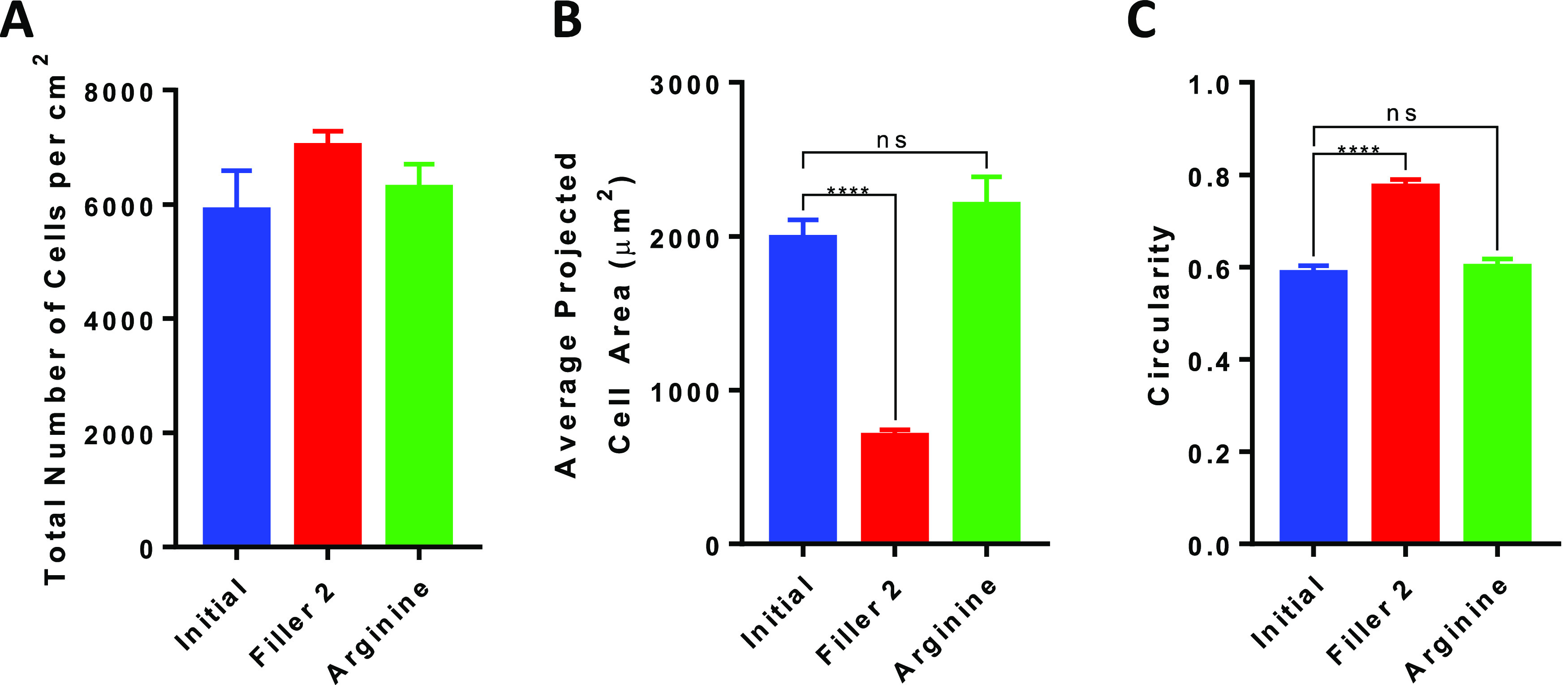
Reversible cell adhesion induced by the molecular exchange. (A) Total number of cells per cm^2^ attached on the surface described in Figure 7. (B) Average projected cell area of MC3T3-E1 attached on the surfaces described in Figure 7. (C) Circularity of MC3T3-E1 attached on the surfaces described in Figure 7 (****p < 0.0001).

Finally, exploiting rSAMs’ stimuli-responsive properties, we investigated whether cell adhesion could be reversed by an amphiphile exchange reaction *i.e.*, replacing “binding” GRGDS 3 in the rSAMs with “nonbinding” Filler 2. After adding Filler 2 (100 μM) into the medium of the adhered cells on the MBA-anchored rSAMs (χ_GRGDS3,Filler2_ = 0.25), a dramatic cell habitus transition from a spread-out cell shape to a nonadhesive round shape (65% reduction in average cell area and increase in circularity of the cells) was observed (Figures 7 and 8). In contrast, conducting the exchange reaction on the rSAM instead with Filler 2 but with the guanidine l-arginine, mimicking Filler 2’s amidine functionality, most of the cells remained in the spread-out habitus after 30 min. To investigate whether the detached cells induced by Filler 2’s addition were still viable, the cell culture medium was replaced by a fresh medium and incubation continued for 24 h. As confirmed by the results shown in Figure S8, this led to a reversal of the cell morphology back to an adhesive spread-out cell shape.

## Conclusions

Supported phospholipid bilayers incorporating bioactive ligands are extensively used as experimental models of the extracellular matrix because of their easily tunable architecture, fluidity, biocompatibility, and functionalization. With these unique set of properties, they offer an ideal microenvironment for regulating the growth and differentiation of cells *in vitro*. Still, widespread applications, particularly in regenerative medicine, going beyond their use as experimental models are limited due to shortcomings in terms of long-term stability, stability toward air exposure, and lack of stimuli responsiveness. The goal of this work was to demonstrate a robust supramolecular system in the form of reversible self-assembled monolayers (rSAMs) combining all of the beneficial properties in one platform. We show that the lateral mobility of rSAMs allows the preparation of bioactive surfaces featuring a tunable lipid bilayer-like fluidity, an easily adjustable ligand presentation combined with stimuli-responsive function for cell harvesting. The results in Figures 5 and 6 coincide well with literature reports on the relationship between cell morphology and lateral mobility. For instance, Kocer et al. demonstrated a 50% increase in average adhered human mesenchymal stem cell (hMSC) area on RGD-functionalized DOPC-based SLBs as compared to less mobile DPPC-SLBs.^31^ This suggests that the rSAM platform, with its tunable surface dynamics, can be used as a versatile alternative to SLBs for modulating and studying cell behavior. Furthermore, the results show that rSAMs can be used to reverse cell adhesion in a noninvasive manner. This is quite different from the established enzymatic trypsin cell removal strategy where the focal adhesion forming integrins are cleaved chemically.^49,50^ The rSAM strategy is very mild in comparison to the trypsin protocol as it operates on a recognition reaction which is diminished or avoided by just removing the recognition sites on the surface by an exchange reaction. The rSAM approach for adhering and detaching cells is therefore causing much less cell stress as the cells do not need to resynthesize the bond-broken integrins for further adhesion as they need to do in the trypsin approach.

Apart from these dynamic properties, the rSAM platform is flexible and can be expanded to include a variety of functionalities. This combination may be useful for matching organ-specific ECM functionality and stiffness, of crucial importance in tissue repair.^23^ In summary, we have demonstrated a versatile tool to study and control cell adhesion and differentiation, offering new interesting cell and tissue engineering perspectives.

## Materials and Methods

### Preparation of rSAMs on Gold-Coated Well Plates and Coverslips

Gold-coated 24-well cell culture plates were prepared as previously reported,^43^ whereas vacuum-packed gold-coated coverslips (*d* = 10 nm) were obtained from Substrata Thin Film Solutions (Kitchener, ON, Canada). The slips were incubated immediately after exposure to ambient atmosphere with 1 mM MBA ethanol solution or 5 μM MDSA in EtOH/water (1/1) for at least 24 h, in the dark, at room temperature. The surfaces were then rinsed with ethanol, dried under a nitrogen stream, and stored in N_2_, in the dark. Prior to cell culture studies, the modified surfaces were rinsed with pH 8 HEPES buffer (0.01 M, pH 8) and subsequently immersed into pH 8 HEPES buffer containing GRGDS 3 and Filler 1 or 2, χ_GRGDS3_ = 0, 0.10, 0.25 (total concentration: 50 μM) at ambient conditions overnight. The amphiphile solution was discarded, and the wells were rinsed three times with pH 8 HEPES buffer.

### Cell Attachment Assay Using Gold-Coated Well Plates

MC3T3-E1 cells were cultured as previously reported and used after a minimum of four passages.^51^ The cells were seeded onto the surfaces prepared above at a density of 1 × 10^4^ cells/cm^2^ and cultured at 37 °C under a humidified atmosphere of 5% CO_2_ for 5 h in an incubator (Heracell CO_2_ cell incubator, Kendro Laboratory, Germany). For cell detachment experiments, 100 μM solutions of Filler 2 or l-arginine were added to the wells and incubated under the same conditions as above. Cell morphology was recorded at different time intervals in a microscope (Olympus CKX41, Olympus Life Science Solutions, Bartlett) equipped with a digital camera for image documentation.

For labeling of the cell cytoskeleton, the culture medium was removed, the samples washed with PBS, and the cells were then fixed using a 4% paraformaldehyde and 1 mM CaCl_2_ solution in PBS. After 15 min, the slides were washed two times with PBS and incubated for 10 min with 0.4% triton-X and 1 mM CaCl_2_ in PBS at room temperature and washed twice with PBS. Subsequently, the cells were labeled with FITC-phalloidin for 1.5 h. After staining, the samples were washed three times with PBS. Analyses and imaging were performed with a fluorescence microscope (Olympus CKX41, Olympus Life Science Solutions, Bartlett).

### Statistical Analysis

Cell culture experiments were based on a minimum of three independent seeding experiments. Average projected cell area and circularity were quantified by analyzing a minimum of 100 cells using a microscope equipped with a digital camera and image processing using ImageJ software. The cell area was estimated based on actin coverage, whereas circularity was calculated using eq 1.
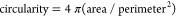
1In all figures, the values are given as mean ± standard deviation. Statistical analyses were performed using GraphPad Prism 7.0. For normally distributed data with equal variances, one-way ANOVA with Tukey’s multiple comparison test was used. A *p*-value < 0.05 was considered significant.

### Assay for Double Labeling Using Phospho-Paxillin and F-Actin

MC3T3-E1 cells were seeded onto the rSAMs-coated coverslips at a density of 1 × 10^4^ cells/cm^2^ and cultured at 37 °C under a humidified atmosphere of 5% CO_2_ for 5 h. Then, the cells were fixed for 15 min with cold 4% paraformaldehyde. After rinsing three times with PBS, the samples were blocked with 1% bovine serum albumin (BSA) in 0.05% Triton-X in PBS. FAs were stained by incubation with a primary Rabbit a-Phospho-paxillin (Tyr118) antibody (Cell signaling, #69363S) diluted 1:600 in 1% BSA + 0.05% Triton-X in PBS at 4 °C overnight. Then, the samples were washed with PBS, 3 × 5 min, and stained with a secondary donkey a-Rabbit AF488 conjugated antibody (Jackson ImmunoResearch (Code nr: 711-546-152) diluted 1:200 times, Cambridge, UK) in 1% BSA + 0.05% Triton-X in PBS for 60 min at rt. The samples were rinsed three times with PBS and then labeled with AF568-conjugated phalloidin (Invitrogen cat # A12380, 5 units/mL, diluted, Waltham) in 1% BSA + 0.05% Triton-X in PBS. After rinsing with PBS, the samples were stained with DAPI for 10 min and rinsed again two times with 0.05% Triton-X in PBS. The samples were mounted and coverslipped in antifade solution (Fluoroshield, Abcam, Cambridge U.K.) for analyses and imaging with an epi-fluorescence microscope (Olympus IX73, Olympus Life Science Solutions, Bartlett) equipped with a DP80 detector (Olympus Life Science Solutions, Bartlett) and 20 × objective. The analysis was performed using an ImageJ-based macro to measure the area and intensity of phospho-paxillin immunofluorescence. The phalloidin labeling was used to define cell area and cell morphology and to evaluate the condition of individual cells. The number of cells was identified automatically from the DAPI staining. Quantification and illustration of raw data were performed using Excel and GraphPad Prism.

## Supporting Information Available

Reagents; apparatus and methods; synthesis of GRGDS 3; and supporting characterization data (PDF)

## Notes

B.S., S.Y.Y., and Y.S. are coinventors on a patent covering the concept presented in this report. The remaining authors declare no competing interests.
