# Supplementary material for: Reversible Self-Assembled Monolayers with Tunable Surface Dynamics for Controlling Cell Adhesion Behavior
Source: ACS Appl Mater Interfaces. 2022 Sep 8;14(37):41790–9. doi: 10.1021/acsami.2c12029 (PMC9501787; doi:10.1021/acsami.2c12029)
Supplement: Supplementary file 1 — am2c12029_si_001.pdf [file am2c12029_si_001.pdf]

Supporting Information

Reversible Self-Assembled Monolayers with Tunable Surface Dynamics for Controlling of Cell Adhesion Behavior

Sing Yee Yeung[1†§], Yulia Sergeeva,[1†] Guoqing Pan[1,2], Silvia Mittler,[3] Thomas Ederth,[4] Tommy Dam,[5] Peter Jönsson,[5] Zahra El-Schich,[1] Anette Gjörloff Wingren,[1] Adam Tillo,[1] Sabrina Hsiung Mattisson,[6] Bo Holmqvist,[6] Maria M. Stollenwerk[1] and Börje Sellergren[1]*

*Corresponding author. Email: borje.sellergren@mau.se

1. Department of Biomedical Sciences and Biofilms-Research Center for Biointerfaces (BRCB), Faculty of Health and Society, Malmö University, 205 06 Malmö, Sweden.

2. Institute for Advanced Materials, School of Materials Science and Engineering, Jiangsu University, Zhenjiang, Jiangsu, 212 013, China

3. Department of Physics and Astronomy, University of Western Ontario, 1151 Richmond Street, London, Ontario, Canada, N6A 3K7

4. Division of Biophysics and Bioengineering, Department of Physics, Chemistry and Biology (IFM), Linköping University, 581 83 Linköping, Sweden

5. Division of Physical Chemistry, Department of Chemistry, Lund University, 221 00 Lund, Sweden

6. ImaGene-iT AB, Medicon Village, Scheelevägen 2, 223 81 Lund, Sweden.

* Email: borje.sellergren@mau.se

§ Present address: PYC Therapeutics, 6 Verdun Street, Nedlands, WA 6009, Australia

This PDF file includes:

Supplementary Text
Scheme S1
Figs. S1 to S7
Table S1
Reference 1

Reagents

All solvents were purchased from Acros Organics (Geel, Belgium) unless otherwise stated. Ethanol (99.5%) was obtained from CCS Health Care (Borlänge, Sweden). GRGDS 4 was purchased from China Peptides (Shanghai, China). Deionized water was used for the chemical synthesis. Type I ultrapure water was obtained with a Thermo Scientific Barnstead NANOpure Diamond Water Purification Systems to give a minimum resistivity of 18.2 MΩ cm^{-1}. All other reagents were purchased from Sigma Aldrich (Sweden) or Merck (Sweden) and used as supplied unless otherwise stated. pH 8 HEPES buffers (0.01 M) were prepared from HEPES ((4-(2-hydroxyethyl)-1-piperazineethanesulfonic acid) and pH 9 borate buffers (0.01 M) were prepared from boric acid. Filler 1 and 2 were synthesized as previously reported.[1] The amphiphile stock solutions (2.5 mM) were prepared in 5 % ethanolic pH 8 HEPES buffer. The MC3T3-E1 cell line (mouse, 99072810) was purchased from Sigma-Aldrich (Sweden).

Apparatus and methods

HPLC analysis was carried out on a Waters 2695 Alliance HPLC system equipped with autosampler, inline degasser, Waters 2996 PDA detector and MassLynx 4.0 software, using a Phenomenex Luna C18(2) column (4.6 mm (i.d.) x 150 mm, 5 µm, 110 Å) and a guard column (4.6 x 20 mm) at ambient temperature. The mobile phase, as indicated in the procedure (vide infra), was pumped at a flow rate of 1.0 mL min^{-1}.

IRAS measurements were made with a Bruker Vertex70 spectrometer, using a custom reflection accessory operating at an incidence angle of 85°, and a liquid nitrogen-cooled "narrow-band" MCT detector (750 to 5000 cm^{-1}, EG&G Judson J15D14-M200). 300 scans were collected at 2 cm^{-1} resolution for each spectrum. A deuterated hexadecanethiol ($HS(CD_2)_{15}CD_3$) SAM on gold was used to record the background spectrum.

Proton and carbon nuclear magnetic resonance spectra were recorded using an Agilent (Varian, location) Mercury 400 MHz instrument operating at 400 or 101 MHz and evaluated using Mestre Nova software. Chemical shifts (δ) are reported in parts per million (ppm) with respect to tetramethylsilane (TMS) using the manufacturer's indirect referencing method. All chemical shifts are quoted on the δ scale in ppm using residual solvent as the internal standard. (^{1}H NMR: $CDCl_3$ = 7.26, CD_3OD = 4.87; DMSO-d_6 = 2.50 and ^{13}C NMR: $CDCl_3$ = 77.0; CD_3OD = 49.0; DMSO-d_6 = 39.5). Coupling constants (J) are reported in Hz with the following splitting abbreviations: s = singlet, d = doublet, t = triplet, q = quartet, quin = quintet, and m = multiplet. Low resolution mass spectra (LRMS) were conducted using a Waters ZQ2000 MS system with 2795 LC and 2996 PDA.

The FRAP measurements were conducted on a customised Nikon Eclipse Ti fluorescence microscope operating in TIRF mode. The microscope was equipped with a 100x oil immersion objective (Plan Apo total internal reflection fluorescence, NA = 1.49; Nikon, Tokyo, Japan) and the images were acquired with a Photometrics Prime 95B sCMOS camera (Tucson, AZ). The E2-FAMs were illuminated with an Oxxius LBX diode laser (Lannion, France) operating at 488 nm. During the bleaching process, a convex lens was placed in the beam path to focus the incoming light onto an approximately 10 µm wide circular region to bleach the fluorophores. The recovery process was recorded by taking images of the samples at 5 s intervals, yielding a recovery curve. The diffusion constant and immobile fraction were obtained from the recovery curve by fitting the

intensity values over time using a MATLAB-written program as described in detail by Jönsson et al. (ref: doi: 10.1529/biophysj.108.134874).

GRGDS-functionalized amidine 3

GRGDS-functionalized amidine 3 was synthesized from GRGDS 4 and azide-terminated amidine 5 based on a modified protocol as previously reported (Scheme S1).[1] Amidine azide precursor 5 (20 mg, 0.036 mmol, 1 eq), GRGDS 4 (19 mg, 0.04 mmol, 1 eq), sodium ascorbate (21 mg, 0.1 mmol, 3 eq) and copper (II) sulphate (5 mg, 0.02 mmol, 0.6 eq) in water/2-butanol/MeOH (1:2:1, 1 mL) was sonicated and stirred at room temperature for 4 hrs. The reaction mixture was concentrated in vacuo and purified using C18 prep chromatography. The purified fractions were then concentrated *in vacuo* at 30°C and the residual water was lyophilized to give the TFA salt of sialic acid terminated amphiphile 2 as an amorphous white powder (18 mg, 54 %).

HPLC (C-18 column, mobile phase: 10% - 90 % ACN (0.1% TFA) in water (0.1% TFA) (0-15 mins)): k=4.3. ^{1}H-NMR (400 MHz, CD3OD) δ 8.41 (s, 1H), 7.77 (d, *J* = 8.9 Hz, 2H), 7.11 (dd, *J* = 7.7, 2.8 Hz, 4H), 6.80 (d, *J* = 8.7 Hz, 2H), 4.62 – 4.57 (m, 2H), 4.47 (t, *J* = 4.1 Hz, 1H), 4.39 (dd, *J* = 7.7, 5.7 Hz, 1H), 4.09 (dd, *J* = 8.8, 4.1 Hz, 4H), 3.92 (t, *J* = 6.5 Hz, 4H), 3.64 – 3.54 (m, 7H), 3.20 (s, 2H), 2.88 (dd, *J* = 17.2, 5.9 Hz, 1H), 2.77 (t, *J* = 6.9 Hz, 3H), 1.94 (s, 1H), 1.87 – 1.63 (m, 8H). LRMS (m/z): $[(M+2H)/2]^+$ calcd for $C_{49}H_{75}N_{13}O_{14}^{2+}$, 535, found 535; $[(M+H)]^+$ calcd for $C_{49}H_{74}N_{13}O_{14}^{2+}$, 1069, found 1069.

Sample preparation protocol for the FRAP experiments

The coverslips coated with gold layer (10 nm, Corning Inc.) were incubated with solution of MBA (1 mM, EtOH) or MDSA (5 μM, EtOH/H_2O 1:1) overnight. The substrates were rinsed with copious amount of ethanol and dried under a nitrogen stream. The MBA-coated coverslips were immersed in the solution of amidines ($\chi_{GRGDS3,Filler2} = 0.10$, total concentration = 50μM) doped with 1% of E2-FAM (Figure S6) overnight. Before the FRAP measurements, the samples were rinsed three times with HEPES buffer (0.01 M, pH 8) dried quickly with nitrogen and then re-hydrated in HEPES buffer.

References

1 Yeung, S. Y. *et al.* Reversible Self-Assembled Monolayers (rSAMs): Adaptable Surfaces for Enhanced Multivalent Interactions and Ultrasensitive Virus Detection. *ACS Central Science* **3**, 1198-1207, doi:10.1021/acscentsci.7b00412 (2017).

Scheme S1.

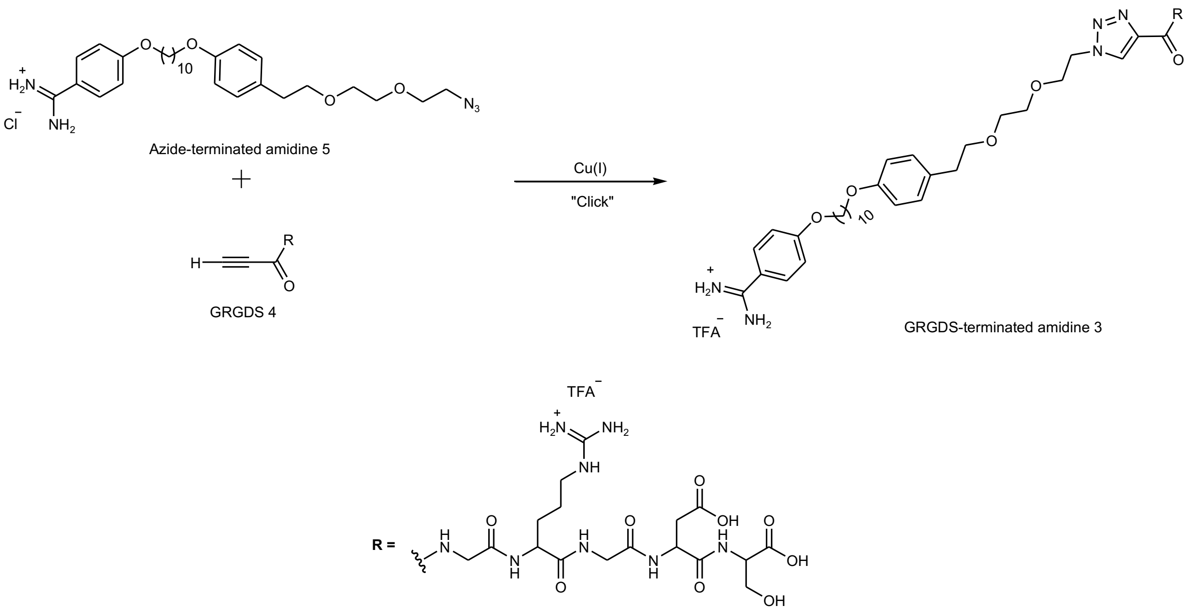

Azide-terminated amidine 5
GRGDS 4
Cu(I)
"Click"
GRGDS-terminated amidine 3
R =

Fig. S1.

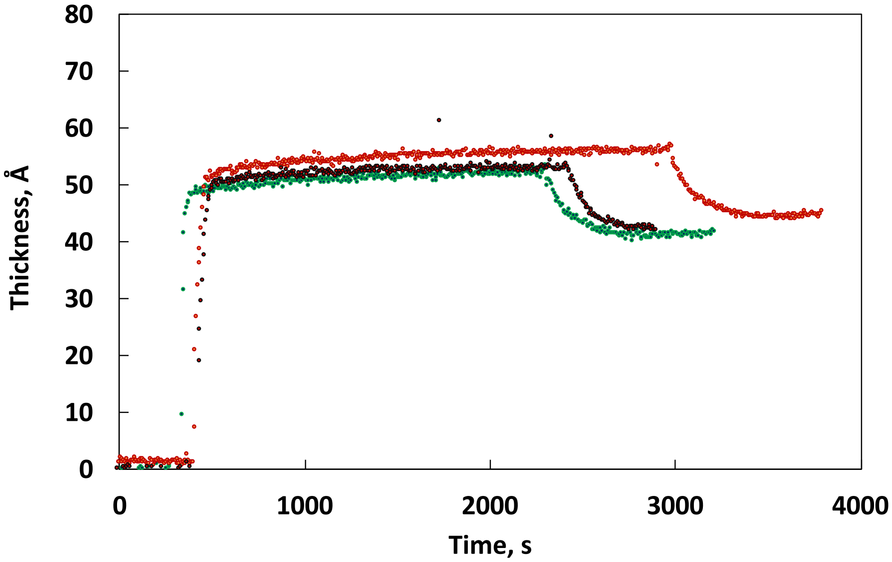

A.

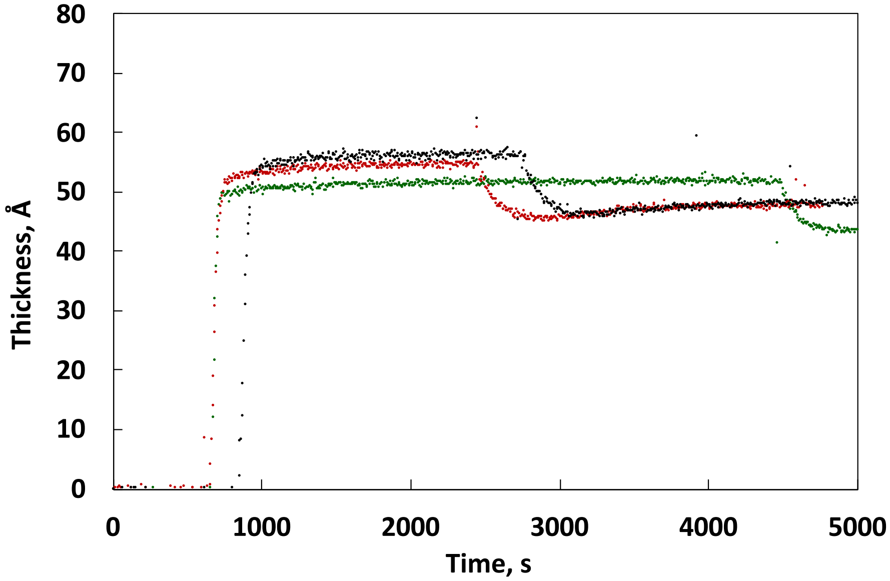

B.

Figure S1. Film thickness, estimated by *in situ* ellipsometry, versus time upon addition of Filler 2 (C=50 μM) in pH 8 HEPES buffer solution (0.01 M) with varying mole fractions of GRGDS 3 with χ_{GRGDS3} = 0 (green trace), 0.1 (red trace) or 0.25 (black trace) to MBA (A) or MDSA (B) SAMs on gold. Final thicknesses measured after rinsing in HEPES pH 8-buffer were respectively in A: 41, 45, 43 Å and in B: 43Å, 48Å, 48Å. As a comparison the molecular end to end distance of the extended forms of Filler 2 and GRGDS were ca 39Å and 47Å. The theoretical thickness of the mixed rSAMs are hence 39Å (χ_{GRGDS3} = 0); 41Å (χ_{GRGDS3} = 0.10)) and 44Å (χ_{GRGDS3} = 0.25) assuming an extended chain conformation with length axis oriented perpendicular to the surface plane.

Fig. S2.

A.

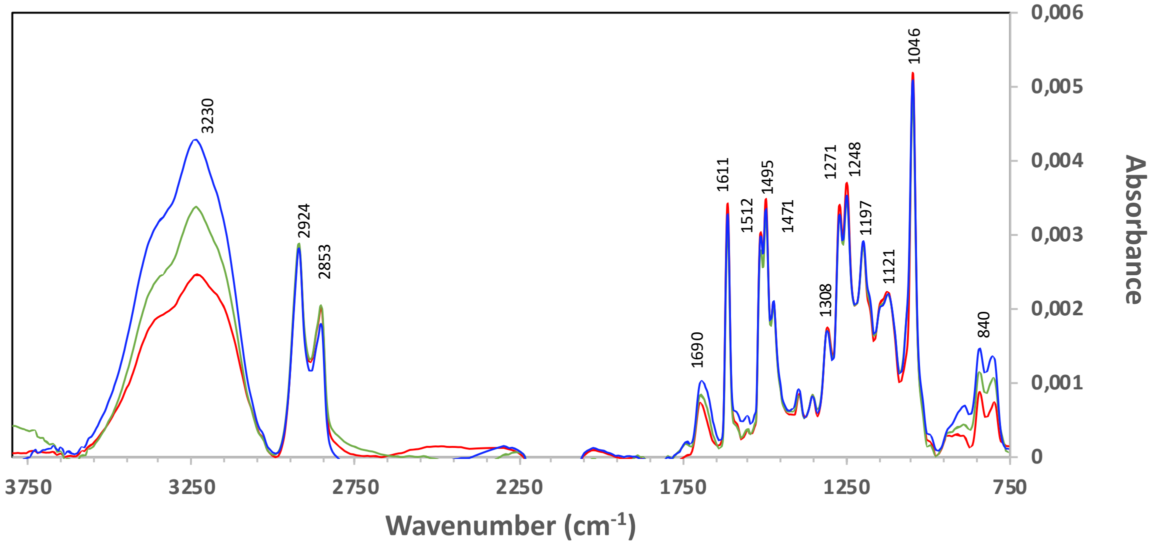

B.

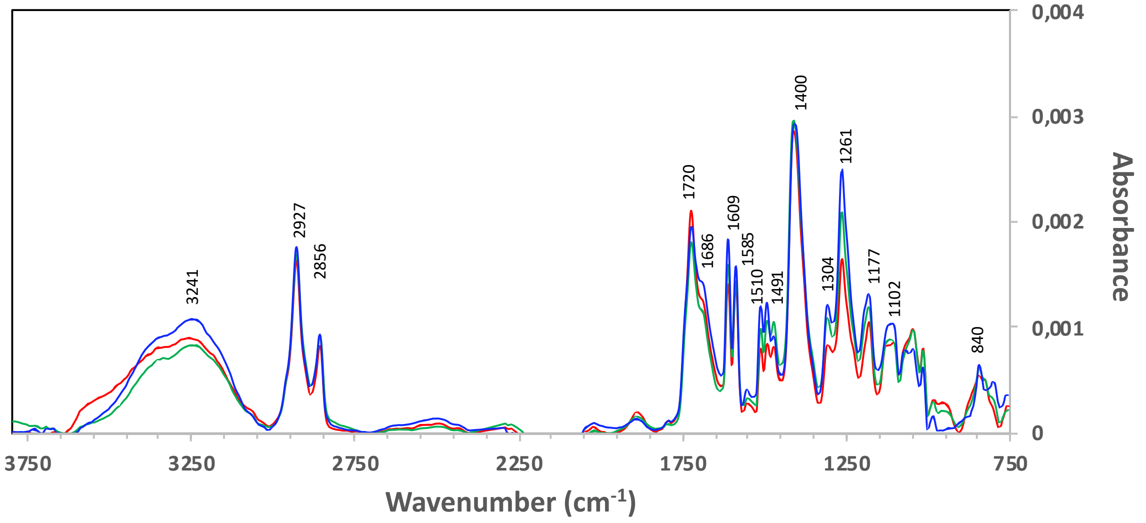

Figure S2. Baseline-corrected IRAS spectra of rSAMs with varying mole fractions of GRGDS 3 in Filler 2, χ_{GRGDS3}: 0 (red trace), 0.1 (green trace), 0.25 (blue trace) on either A) MDSA or B) MBA SAMs.

Fig. S3.

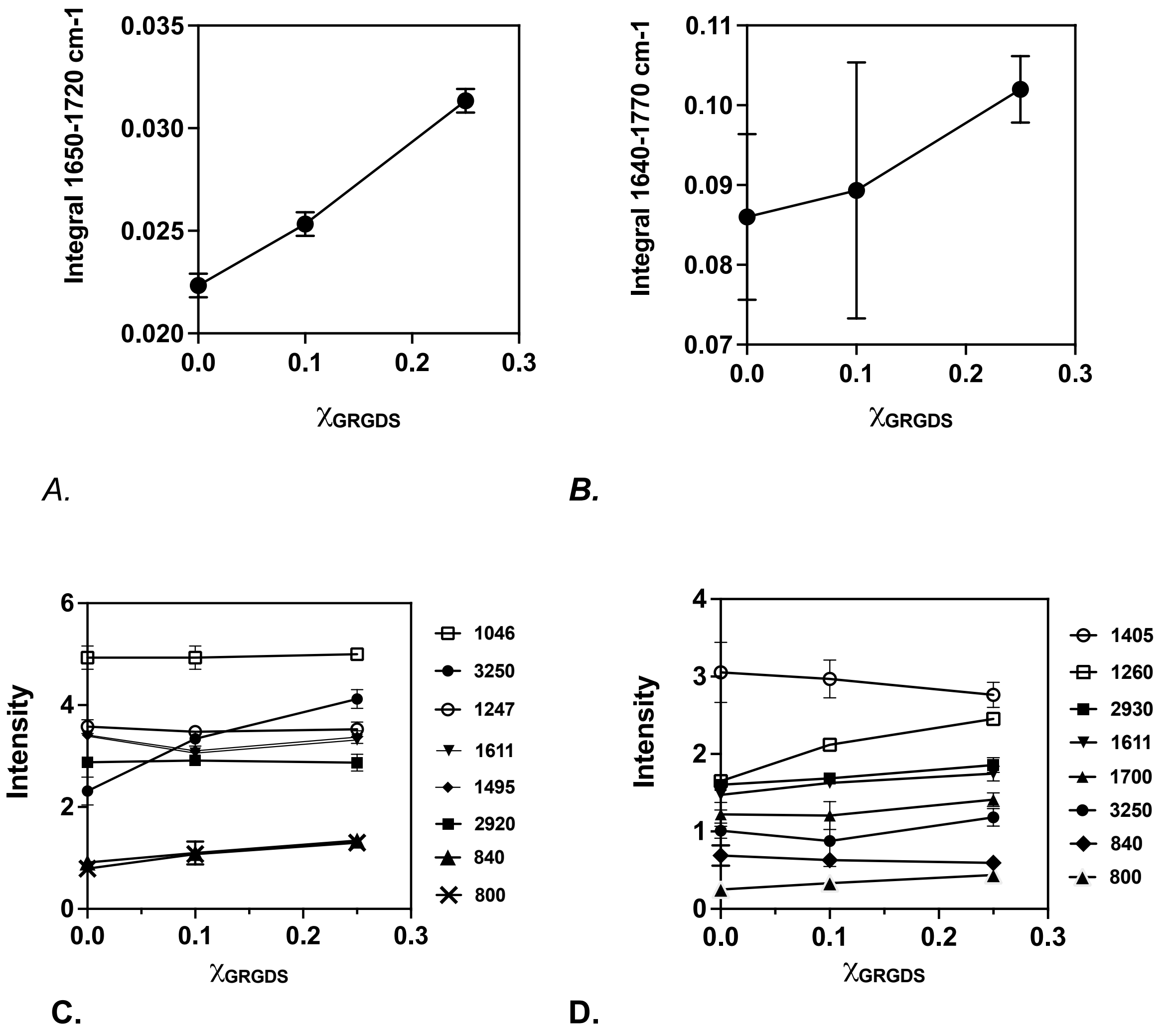

Figure S3. Integrals (A,B) and intensities (C,D) of diagnostic bands from IRAS spectra (n=3) of rSAMs with varying mole fractions of GRGDS 3 (χ_{GRGDS3}) in Filler 2 using MDSA (A,C) or MBA (B,D) as anchor SAMs.

Fig. S4.

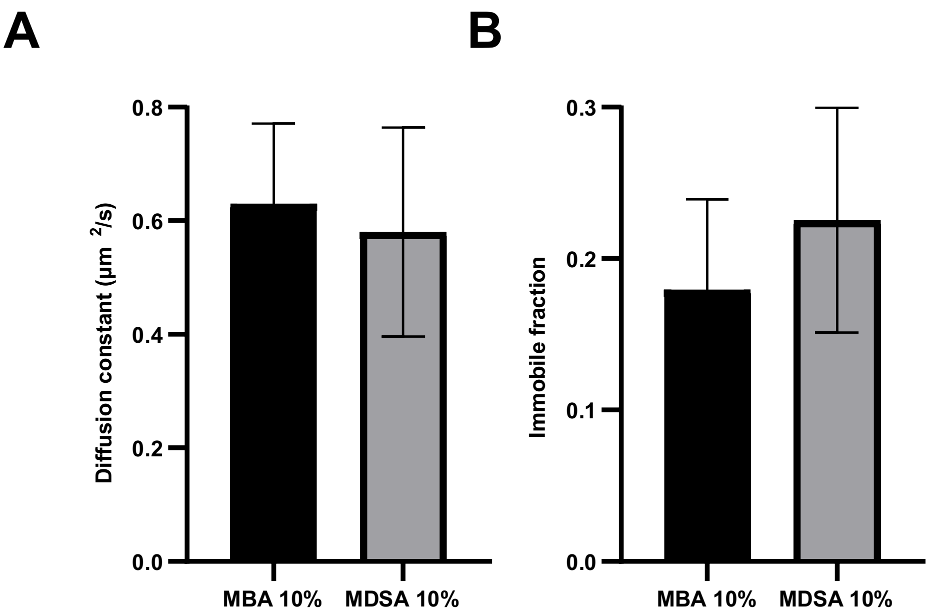

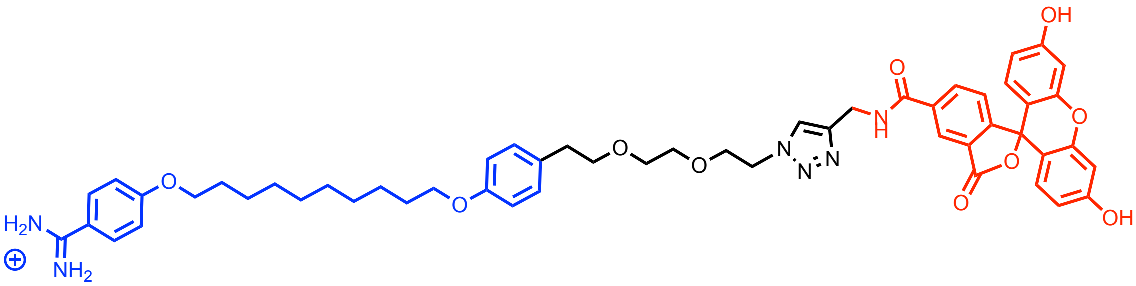

E2-FAM

Figure S4. The diffusion constant (A) and the immobile fraction (B) of E2-FAM (see structure) in MBA or MDSA SAMs on gold ($\chi_{GRGDS3} = 0.1$) measured by FRAP (mean ± s.d. from $n_{MBA} = 11$ and $n_{MDSA} = 15$ measurements)

Fig. S5.

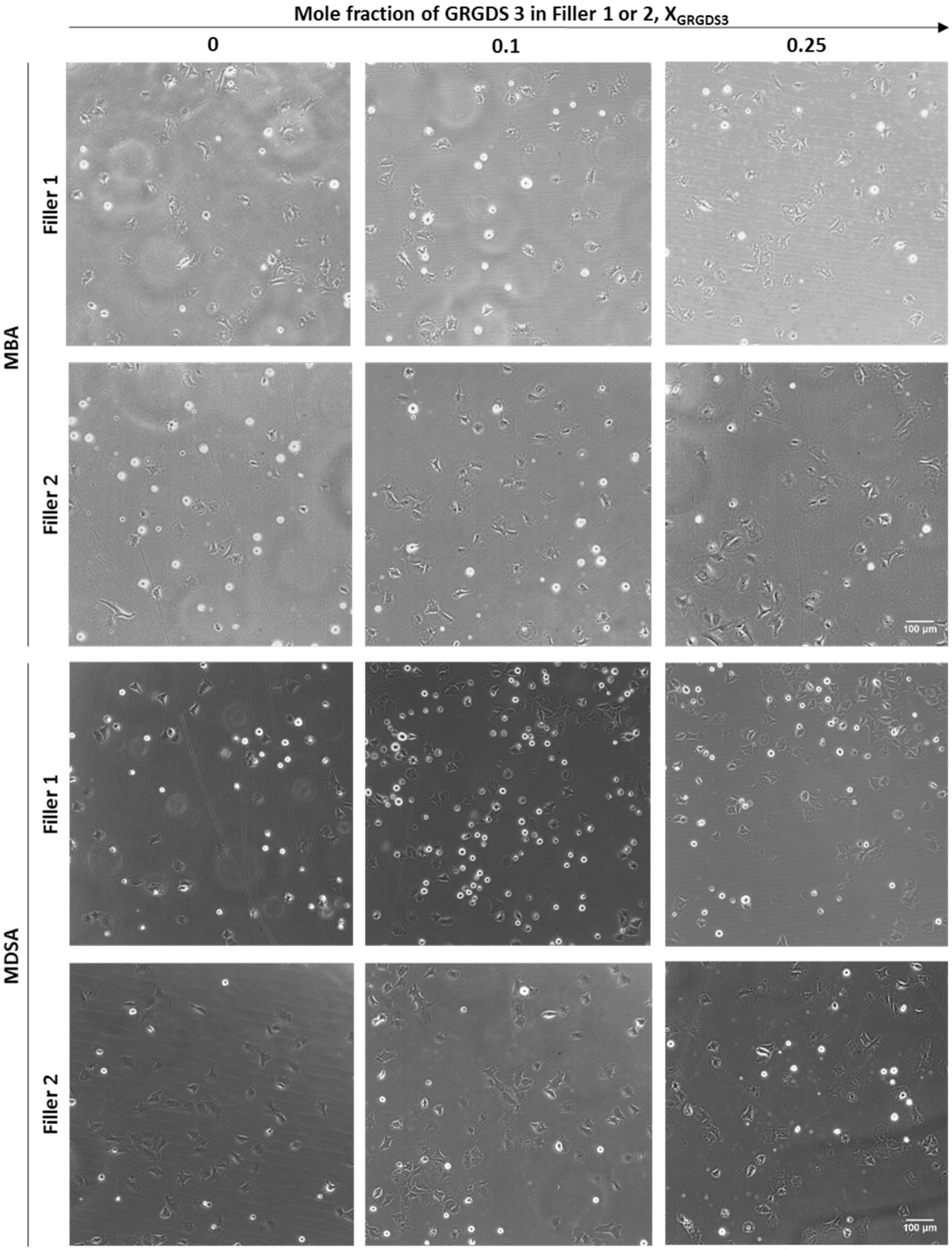

Figure S5. Brightfield micrographs of MC3T3-E1 adhered on surfaces modified with varying mole fractions of GRGDS 3 in either Filler 1 or 2, χ_{GRGDS3} on either MBA or MDSA SAMs.

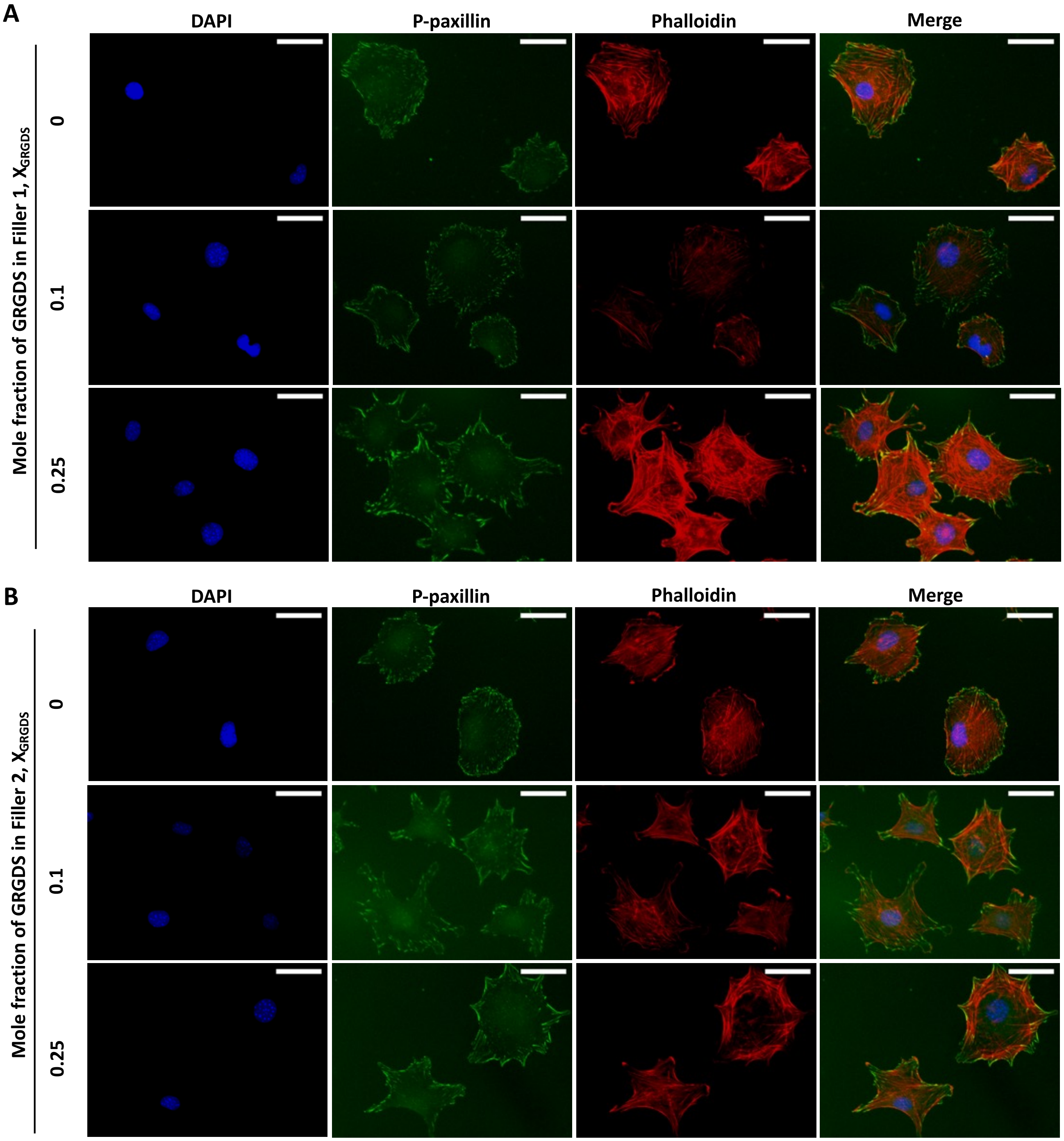

Figure S6. Double immunofluorescence labelling. Representative images illustrate the effect by GRGDS 3 density and filler amphiphiles (A: Filler 1, B: Filler 2) on morphology of MC3T3-E1 cells adhered to rSAMs anchored on MBA-SAMs. The labelling used to visualize the cells are: nucleus (DAPI: blue), focal adhesions (phospho-paxillin: green), actin filaments (phalloidin: red). The images were recorded 5 h after seeding. Scale bars = 50 µm.

Fig. S7.

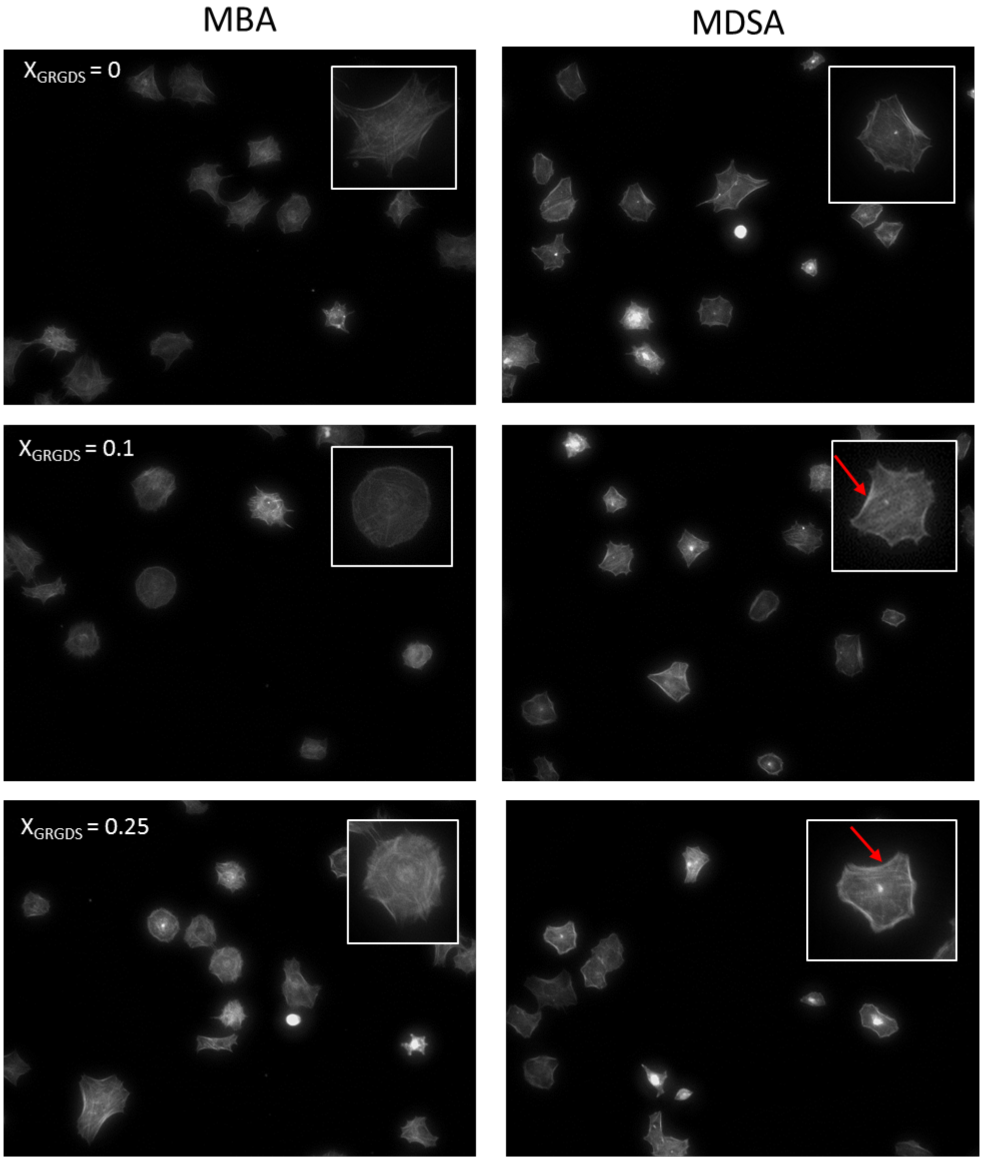

Figure S7. Fluoresence micrographs of FITC-phalloidin stained MC3T3-E1 adhered on surfaces modified with varying mole fractions of GRGDS 3 in Filler 1, χ_{GRGDS3} on either MBA or MDSA SAMs.

Fig. S8.

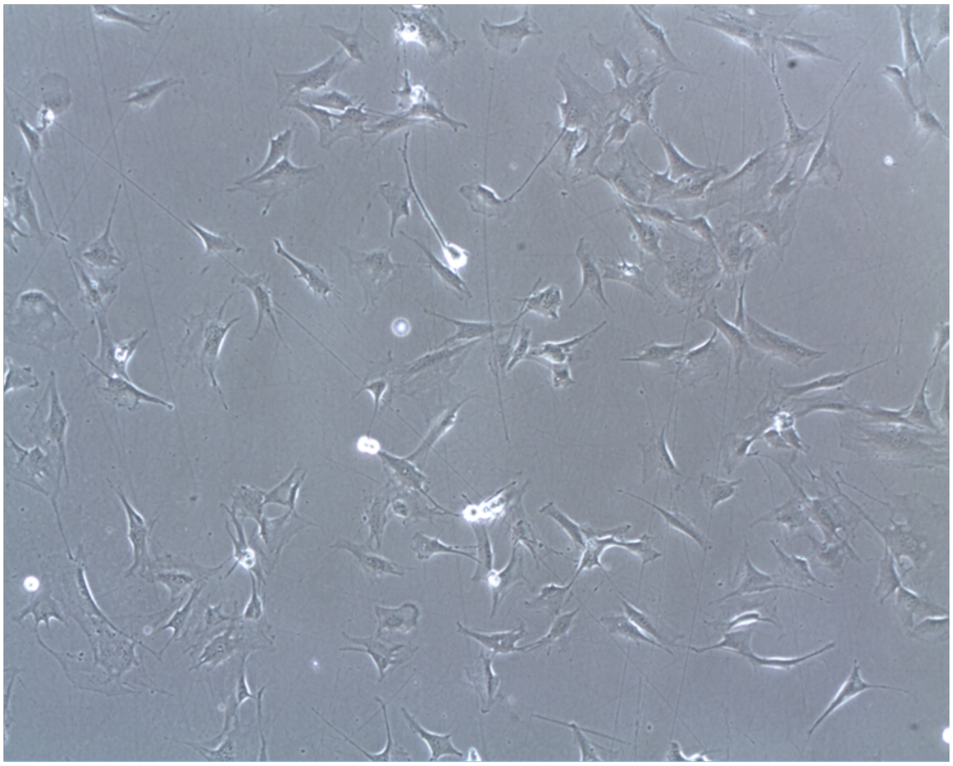

Figure S8. Brightfield micrographs of MC3T3-E1 after 100 μM exposure to Filler 2.

Table S1.

Table S1. Positions and spectral mode assignments of the IR bands of a mixed rSAM of GRGDS 3 (χ=0.25) and Filler 2 on SAMs of MBA or MDSA.

**Mode assignment**	**MBA-SAM**		**MDSA-SAM**	
	MBA	GRGDS 3	MDSA	GRGDS 3
NH_2, N-H, O-H stretch (asym)	-	3230	-	3230
CH_2, C–H stretch (asym)	-	2927	2924	2924
CH_2, C–H stretch (sym)	-	2856	2853	2853
Amidinium, N-C=N stretch (asym), C=O stretch	1720	-	-	1690
Amide I (C=O stretchstretch)	-	1690	-	1677
Aromatic C=C stretch (‖1,4 axis)		1609/1510/1491	-	1611/1512/1495
Aromatic C=C stretch (‖1,4 axis)	1585	-	-	-
Amide II (NH bendbend)	-	1550	-	1550
COO^- stretch (sym), COH deform	1400	-	-	-
Aromatic ethers, aryl-O stretch (asym)	-	1261	-	1248
SO_4 – stretch (asym)	-	-	1271	-
SO_4 – stretch (sym)		-	1046	-
Aromatic C–H bending (out of plane)	-	840	-	840
Amide V (NH out of plane bending)	-	800	-	800
